# Transgenerational Sex-dependent Disruption of Dopamine Function Induced by Maternal Immune Activation

**DOI:** 10.3389/fphar.2022.821498

**Published:** 2022-02-08

**Authors:** Michele Santoni, Roberto Frau, Marco Pistis

**Affiliations:** ^1^ Department of Biomedical Sciences, Section of Neuroscience and Clinical Pharmacology, University of Cagliari, Cagliari, Italy; ^2^ “Guy Everett” Laboratory, University of Cagliari, Cagliari, Italy; ^3^ Neuroscience Institute, Section of Cagliari, National Research Council of Italy (CNR), Cagliari, Italy; ^4^ Unit of Clinical Pharmacology, University Hospital, Cagliari, Italy

**Keywords:** dopamine, electrophysiology, sex-differences, transgenerational transmission, maternal immune activation

## Abstract

Several epidemiological studies suggest an association between maternal infections during pregnancy and the emergence of neurodevelopmental disorders in the offspring, such as autism and schizophrenia. Animal models broadened the knowledge about the pathophysiological mechanisms that develop from prenatal infection to the onset of psychopathological phenotype. Mounting evidence supports the hypothesis that detrimental effects of maternal immune activation might be transmitted across generations. Here, we explored the transgenerational effects on the dopamine system of a maternal immune activation model based on the viral mimetic polyriboinosinic-polyribocytidilic acid. We assessed dopamine neurons activity in the ventral tegmental area by *in vivo* electrophysiology. Furthermore, we studied two behavioral tests strictly modulated by the mesolimbic dopamine system, i.e., the open field in response to amphetamine and the prepulse inhibition of the startle reflex in response to the D2 agonist apomorphine. Second-generation adult male rats did not display any deficit in sensorimotor gating; however, they displayed an altered activity of ventral tegmental area dopamine neurons, indexed by a reduced spontaneous firing rate and a heightened motor activation in response to amphetamine administration in the open field. On the other hand, second-generation female rats were protected from ancestors’ polyriboinosinic-polyribocytidilic acid treatment, as they did not show any alteration in dopamine cell activity or in behavioral tests. These results confirm that maternal immune activation negatively influences, in a sex-dependent manner, neurodevelopmental trajectories of the dopamine system across generations.

## 1 Introduction

A wealth of evidence suggests that exposure to environmental factors during pregnancy increases the risk of developing neuropsychiatric diseases in the offspring. This relationship lies in the negative influences that environmental insults may have on the brain during this critical development period, in which the CNS is particularly responsive to intrinsic (genetic) or extrinsic (environmental) factors. Among the vast number of environmental factors able to elicit detrimental effects on CNS development of the fetus, maternal infections appear to play a primary role in the aetiologies of neurodevelopmental diseases, such as autism and schizophrenia (Miller et al., 2013; Allswede et al., 2020). Although the molecular mechanisms underlying the pathological interaction between a prenatal infection and the developing brain are not fully elucidated yet, there is a broad consensus in considering abnormal cytokine-associated inflammatory events as a critical component in mediating the adverse effects of maternal immune activation (MIA) on the fetal brain.

Animal models have offered considerable insights into understanding the pathophysiological mechanisms that occur and develop from prenatal infection to the onset of psychopathological phenotype (Brown and Meyer, 2018). One of the rodent models with optimal face, construct, and predictive validity for these disorders is based on polyriboinosinic-polyribocytidylic acid (poly (I:C), a double-stranded synthetic RNA. Poly (I:C), when administered at specific stages of rodent pregnancy, like a viral infection, triggers a pathological immune response leading to schizophrenia-like phenotype in offspring (Zuckerman et al., 2003; Meyer and Feldon, 2009; Talukdar et al., 2020; Purves-Tyson et al., 2021). Accordingly, poly (I:C) progenies exhibit a plethora of spontaneous behavioral aberrations relevant to schizophrenia, including deficits in sensorimotor gating, social interaction, and working memory (Meyer, 2013; Ding et al., 2019; Kreitz et al., 2020). In addition, MIA rats exhibit heightened susceptibility to the effects of the psychotomimetic drugs amphetamine and phencyclidine, which are known to elicit positive, negative, and cognitive schizophrenia-like symptoms in healthy humans and laboratory animals (Meyer et al., 2006; Ozawa et al., 2006). Remarkably, these psychotic-like phenotypes are especially underpinned by alterations in the mesolimbic dopamine system, whose abnormalities are considered a final common mechanism in the neuropathogenesis of schizophrenia (Meyer et al., 2008a; Vuillermot et al., 2010). Accordingly, previous studies using MIA as prenatal insults have found abnormalities in the dopamine system in the offspring, such as increased numbers of tyrosine hydroxylase (TH) immunoreactive cells in the VTA and TH-positive terminals in the striatum (Meyer et al., 2008b; Winter et al., 2009; Vuillermot et al., 2010) or dopamine output in striatal slices (Zuckerman et al., 2003) in the lateral globus pallidus and prefrontal cortex (Winter et al., 2009). Our group previously reported that poly (I:C) males, but not females, exhibit behavioral deficits reminiscent of schizophrenia-spectrum disorders, along with alterations in the mesocorticolimbic pathway. Consistently, we reported a marked change of VTA dopamine neuron activity and a higher dopamine release in the NAc (Luchicchi et al., 2016; De Felice et al., 2019; Lecca et al., 2019). Of note, it has been recently demonstrated the involvement of MIA effects not only in the first descendants but also across generations suggesting a transgenerational transmission (Weber-Stadlbauer et al., 2017; Weber-Stadlbauer et al., 2021). These studies showed the comparison among behavioral, neurochemical and transcriptomic changes across generations and the emergence of novel phenotypes. Although the attention on transgenerational transmission is growing, to our knowledge, no study has yet combined sex-specific behavioral and electrophysiological characterization of MIA models across generations. Thus, the purpose of this work is to investigate the transgenerational effects of MIA on the mesolimbic dopamine pathway of the second generation (F2) of male and female rats. We first assessed the impact of poly (I:C) in the F2 by studying the two major representative psychotic-like phenotypes reported in the first generation (F1) offspring (De Felice et al., 2019) and underpinned by an altered mesolimbic dopamine transmission, i.e. the amphetamine-induced hyperactivity and deficits in PPI. In particular, we studied F2 behavioral phenotypes both at baseline and upon the administration of the dopaminomimetics amphetamine and apomorphine, which are known to elicit hyperactivity in the open field (OF) and disruption of PPI, respectively (Devoto et al., 2012; Sagheddu et al., 2021). Furthermore, to detect potential higher susceptibility of Poly I:C-F2 progenies in the dopaminergic responses to dopaminergic agonists, we chose to administer a low dose of amphetamine (AMPH) (0.1 mg/kg, SC) for the OF test and apomorphine (APO) (0.125 mg/kg, SC) for prepulse inibithion (PPI) paradigm. Finally, to better detect the activity of the mesolimbic dopamine function, we assessed dopamine neuron activity by *in vivo* electrophysiology.

## 2 Material and Methods

### 2.1 Animals

**FIGURE 1:**
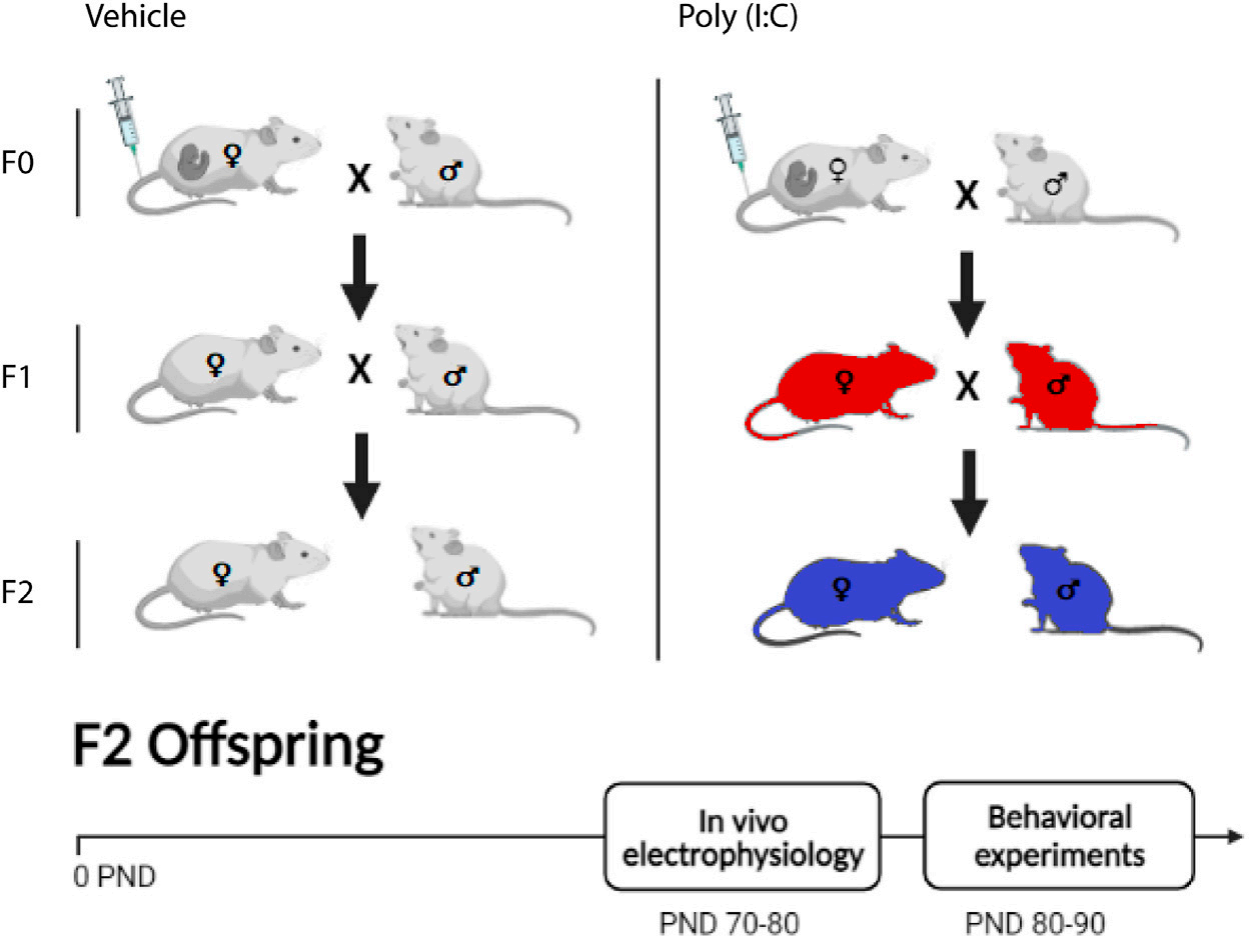
Representation of the experimental protocol. Polyriboinosinic-polyribocytidilic acid [poly (I:C)] treatment during pregnancy of F0 dams consisted in a single i. v. injection of poly (I:C) (4 mg/kg) or vehicle (sterile pyrogen-free saline) at the15th gestational day (GD15). F1 and F2 dams were not treated. In white the F0 generation, treated with poly (I:C) or vehicle. The same color was used both for F1 and F2 that descended from vehicle-treated F0 generation. In red the F1 poly (I:C)-generation, direct descendants from the F0 poly (I:C) insult. In blue, the F2 poly (I:C)-generation, which descends from F1 non-treated generation. *In vivo* electrophysiology recordings were performed between postnatal days (PND) 70 and 80, whereas behavioral experiments between 80 and 90 PND.

Female Sprague Dawley rats (Envigo, Italy) were mated at the age of 3 months. F0 pregnant dams on gestational day (GD) 15 were randomly assigned to receiving either a single injection of poly (I:C) or an equivalent volume of endotoxin-free saline solution in the lateral vein of the tail. Maternal care has not been assessed neither in F0 nor in F1 dams. Animals were housed in groups of 3–4 in standard conditions of temperature (21 ± 1°C) and humidity (60%) under a 12-h-light/-dark cycle (with lights on at 7:00 am) with food and water available ad libitum. All F1 offspring were weaned on the postnatal day (PND) 21 and randomly allocated to behavioral, neurochemical or electrophysiological recordings, which confirm the effectiveness of poly (I:C) treatment (for F1 data refers to De Felice et al., 2019 and to Lecca et al., 2019). In particular, the F1 offspring allocated for breeding were male and female littermates randomly selected 10 days after the completion of behavioral (PPI) testing. F2 offspring were weaned on PND 21 and from PND 70 were either allocated to behavioral testing or electrophysiological recordings (see Supplementary Material) (Figure 1). Experiments were approved by the Animal Ethics Committees of the University of Cagliari and were carried out in accordance with the European Directive on the protection of animals used for scientific purposes (2010/63/EU).

### 2.2 Drugs and Treatments

High molecular weight (HMV) poly (I:C) was purchased from InvivoGen. Poly (I:C) was dissolved in endotoxin-free saline solution, (4.0 mg/kg) and injected in the lateral vein of the tail of F0 pregnant dams. To assess the efficacy of poly (I:C) injection, all pregnant rats were weighed for the first 3 days after the administration of either poly (I:C) or endotoxin-free saline to evaluate potential weight loss as underlined by previous investigations (Zuckerman et al., 2003).

Apomorphine (APO, 0.125 mg/kg) (Merck Sigma-Aldrich) was dissolved in a solution containing 0.9% NaCl with 0.1 mg/ml ascorbic acid (pH 7.2) to prevent oxidation. D-Amphetamine sulfate (AMPH, 0.1 mg/kg) (Merck Sigma-Aldrich) was dissolved in a solution containing 0.9% NaCl. APO and AMPH were administered subcutaneously and intraperitoneally in an injection volume of 1 and 2 ml/kg, respectively.

### 2.3 Prepulse Inhibition of Startle Reflex

Prepulse Inhibition (PPI) of Startle Reflex Startle and PPI were performed as previously described by (Frau et al., 2019) with slight modifications. The apparatus used for the detection of startle reflexes (Med Associates, St Albans, VT) consisted of 4 standard cages placed in sound-attenuated chambers with fan ventilation. Each cage consisted of a Plexiglas cylinder of 9-cm diameter mounted on a piezoelectric accelerometric platform connected to an analog-digital converter. Two separate speakers conveyed background noise and acoustic bursts, each one properly placed to produce a variation of sound within 1 dB across the startle cage. Both speakers and startle cages were connected to a main PC, which detected and analyzed all chamber variables with specific software. Before each testing session, acoustic stimuli and mechanical responses were calibrated *via* specific devices supplied by Med Associates. On the testing day, each rat was placed in the cage for a 5-min acclimatization period consisting of 65-dB white noise background, which continued for the remainder of the session. Each session consisted of three consecutive sequences of trials (blocks).

During the first and third blocks, rats were presented with only 5 pulse-alone trials of 110 dB. In the second block was delivered a pseudorandom sequence of 50 trials, including 12 pulse-alone trials; 30 trials of pulse preceded by 70-, 74-, or 78-dB prepulses (10 for each level of prepulse loudness); and 8 no-stimulus trials, where only the background noise was delivered. Pulse and prepulse durations were set at 40 and 20 ms, respectively. Inter-trial intervals were selected randomly between 10 and 15 s, while the inter-stimulus intervals were set at 100 milliseconds. The startle response was based on the first positive wave that meets the minimum wave criteria and determined as the mean startle amplitude of the pulse-alone trials relative to the second block. Startle habituation across the two halves of the second block was evaluated as a percent inter-block ratio using the following formula: (mean startle amplitude for the first half of the second block/mean startle amplitude for the second half of the second block) × 100. Latency to startle was based on the first peak value across pulse-alone trials of the second block. “Arbitrary units” were calculated by the Med Associates apparatus software by proportionally converting the analog voltage signal recorded by the startle sensor (ranging from −10 to +10 V) to a digital unit, within a range of values between –2048 and +2048. The % PPI was calculated only on the values relative to the second block using the following formula: [(mean startle amplitude pulse alone trials mean startle amplitude prepulse + pulse trials)/mean startle amplitude for pulse alone trials] × 100.

### 2.4 Locomotor Activity

Locomotor behaviors were carried out during the light phase in a temperature- and humidity-controlled room with a dimly background light. Testing chambers were placed to avoid any bias due to direct light, dark corners, or shadowed areas. The apparatus consisted of a square arena (40 × 40 cm^2^), surrounded by four 40-cm transparent Plexiglas walls. Each cage had two sets of 16 photocells located at right angles to each other, projecting horizontal infrared beams 2.5 cm apart and 2 cm above the cage floor. At the beginning of the test, rats were placed individually in the center of the arena and behavioral phenotypes were collected every 10 min *via* custom software (Omnitech Digiscan monitoring system). Behavioral phenotypes were assessed for 90 min (30 min of baseline activity and 60 min after amphetamine injection) and included horizontal and vertical activity (counts), and the periphery and center time, calculated as the durations of time spent detected by photocells along the perimeter of the walls or in the center of the arena.

### 2.5 *In Vivo* Electrophysiological Experiments

**FIGURE 2:**
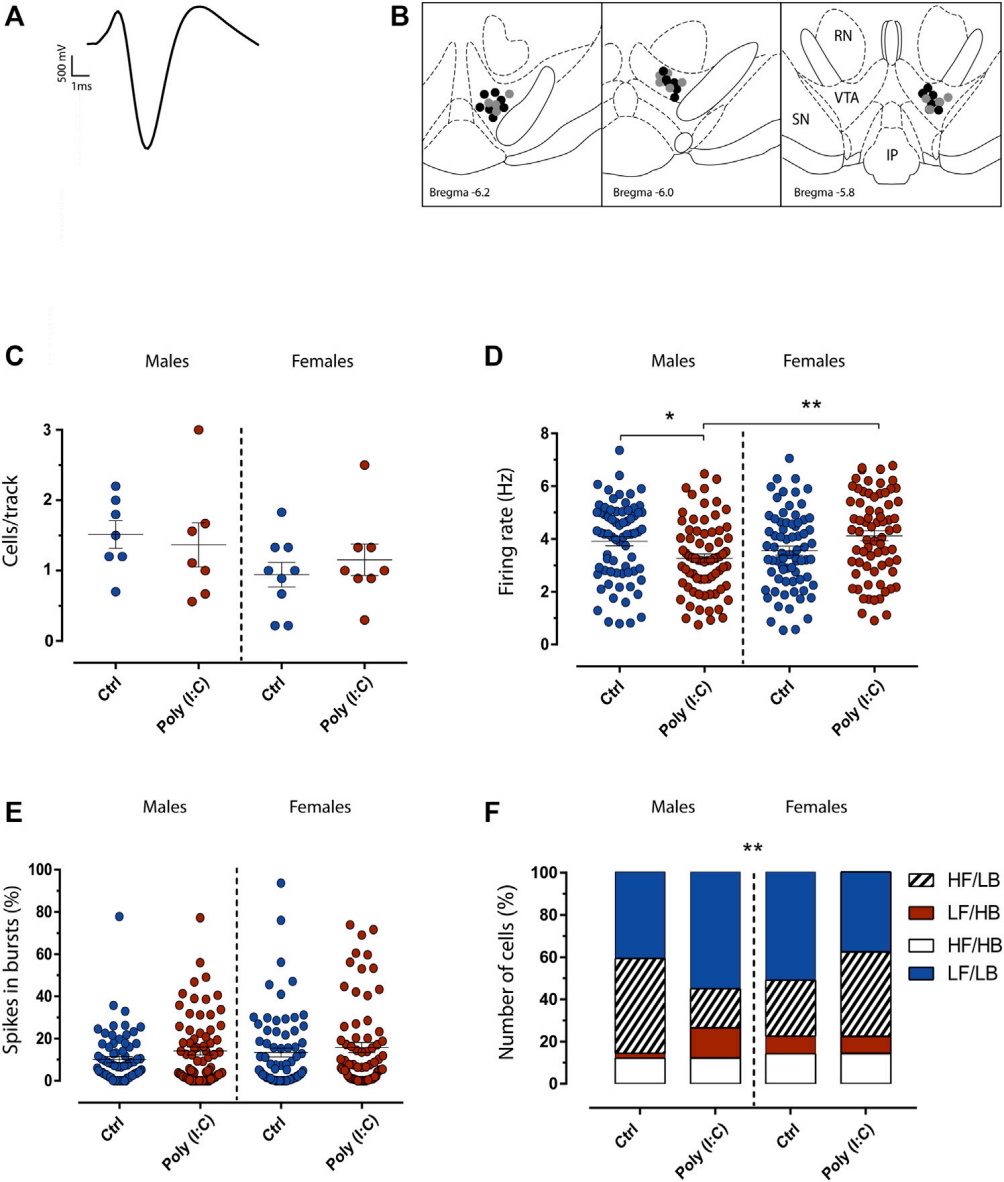
Maternal immune activation transgenerationally dysregulates dopamine neuron firing activity in adult F2 rats. **(A)** The typical broad spike waveform of a dopamine neuron. **(B)** Representative localization of recording sites of VTA putative dopamine neurons in Ctrl (grey dots) and poly (I:C) (black dots) offspring, as verified by histological sections. RN, red nucleus; IP, interpeduncularnucleus, SN, substantia nigra pars reticulata. **(C)** The Scatter plot shows the number of spontaneously active dopamine neurons. **(D)** Scatter plot shows individual dopamine neuron firing rate in Ctrl and Poly (I:C) rats. The scatter plot in **(E)** displays individual percentages of spikes in bursts between Ctrl and Poly (I:C). Bar graphs in **(F)** show the percentage of cells that display a different pattern of discharge. LF (low frequency, firing rate < 4.0 Hz), LB (low bursting, percentage of spikes in bursts < 20%), HF (high frequency, firing rate >4.0 Hz) and HB (high bursting, percentage of spikes in bursts >20%). Data are expressed as means ± SEM. *p < .05.

Rats were anesthetized with urethane 1.3 g/kg, IP. Rats were placed in a stereotaxic apparatus (Kopf, Tujunga, CA, United States) with their body temperature maintained at 37 ± 1°C by a heating pad. The recording electrode was placed above the VTA (5.6–6.2 posterior to bregma, 0.4–0.8 mm lateral to midline, 7.0–8.0 mm from cortical surface; Figure 2B), according to the stereotaxic rat brain atlas of Paxinos and Watson (Paxinos and Watson, 2007). Single unit activity of neurons was recorded extracellularly (bandpass filter 0.1–10,000 Hz) with glass micropipettes filled with 2% Pontamine sky blue dissolved in 0.5 M sodium acetate. Individual action potentials were isolated and amplified through a window discriminator (Neurolog System, Digitimer, Hertfordshire, United Kingdom) and displayed on a digital storage oscilloscope (TDS 3012, Tektronics, Marlow, United Kingdom). Experiments were sampled online and offline with Spike2 software by a computer connected to CED1401 interface (Cambridge Electronic Design, Cambridge, United Kingdom). Dopamine neurons were isolated and identified according to previously described electrophysiological characteristics (Grace and Bunney, 1983; Ungless et al., 2004; Ungless and Grace, 2012). VTA dopamine neurons were recorded only when criteria for identification were fulfilled (firing rate 0.5–10 Hz, duration of action potential ≥2.5 ms, Figure 2A). Bursts were defined as the occurrence of two spikes at interspike interval <80 ms and terminated when the interspike interval exceeded 160 ms.

### 2.6 Statistical Analysis

Data normality and homoscedasticity were preliminarily verified using the goodness-of-fit tests Kolmogorov-Smirnov and Bartlett’s tests. Once assess these ANOVA assumptions, we analyzed the data by one or multiway ANOVAs followed by Tukey’s test for post-hoc comparisons, with Spjøtvoll-Stoline’s correction for unequal n whenever necessary. Chi-square test and *t*-test were applied when necessary. Significance threshold was set at 0.05.

## 3 Results

### 3.1 Transgenerational Effects of MIA on Dopamine Cell Activity and Functionality *In Vivo*

Consistent with our previous studies (Luchicchi et al., 2016; De Felice et al., 2019), Poly (I:C) exposure elicited a significant weight loss in rat dams at 24 h from its administration (vehicle: 10.7 ± 1.3, *n* = 11; poly (I:C): 1.8 ± 1.5, *n* = 12) (*p* < .001, student’s *t*-test) (See Supplementary Figure S1).

The ventral tegmental area (VTA) is a key brain region that plays a crucial role in motivation and reward-related processing (Lammel et al., 2014; Morales and Margolis, 2017). Midbrain dopamine dysfunctions are implicated in several psychiatric disorders, such as schizophrenia (Sonnenschein et al., 2020). We previously demonstrated that MIA impacts electrophysiological properties of midbrain dopamine neurons in F1 male, but not female, offspring (De Felice et al., 2019; Lecca et al., 2019). Therefore, we carried out *in-vivo* single-unit extracellular recordings of VTA dopamine neurons in anesthetized F2 male and female rats to determine whether MIA transgenerationally induced sex-dependent effects on the spontaneous activity of dopamine cells. For these experiments we utilized *n* = 7 vehicle-treated and *n* = 7 poly (I:C)-treated male offsprings, *n* = 9 vehicle-treated and *n* = 8 poly (I:C)-treated female offsprings. Analysis of the number of cells/track (Figure 2C), which is an index that describes the population activity of dopamine neurons, did not reveal any difference (F (1, 27) = 0.62, *p* > .05). On the other hand, a Two-way ANOVA analysis of the firing rate of dopamine cells recorded from male and female rats revealed an interaction between sex and treatment (F (1, 282) = 12.05, *p* < .05). Post hoc analysis (Tukey multiple comparisons test) showed that male offspring displayed a reduction in the mean spontaneous firing rate of VTA dopamine cells recorded from rats exposed to poly (I:C) when compared with control males (Figure 2D), whereas in females the average spontaneous firing rate of VTA dopamine cells did not differ between poly (I:C) and controls (Luchicchi et al., 2016). No change overall was shown for the percent of spikes in bursts (interaction: F (1, 281) = 0.1879, *p* > .05). (Figure 2E). Moreover, VTA dopamine neurons activity could be described following a firing/bursting distribution (Mameli-Engvall et al., 2006). Interestingly, the analysis of population distribution between low frequency (LF, firing rate <4.0 Hz), low bursting (LB, percentage of spikes in bursts <20%) high frequency (HF, firing rate >4.0 Hz) and high bursting (HB, percentage of spikes in bursts >20%), revealed that poly (I:C)-treated males offspring showed more LB/LF cells (39 cells, 54%) compared to poly (I:C)-treated females (26 cells, 37%). (Figure 2F). This is consistent with the difference revealed in the decreased frequency in the poly (I:C)-treated group (Figure 2D) (Chi-square test; *p* < .05).

### 3.2 Behavioral Phenotypes in F2 Male and Female Poly (I:C) Generation

We next investigated whether the sex-dependent impact of MIA on VTA dopamine neuron activity might be accompanied by behavioral alterations in two tests strictly modulated by the mesolimbic dopamine system, i.e., the open field (OF) and the prepulse inhibition of the startle reflex (PPI). For the evaluation of both OF and PPI paradigm, we utilized *n* = 17 vehicle-treated and *n* = 15 poly (I:C)-treated male offsprings, *n* = 14 vehicle-treated and *n* = 14 poly (I:C)-treated female offsprings.

**FIGURE 3:**
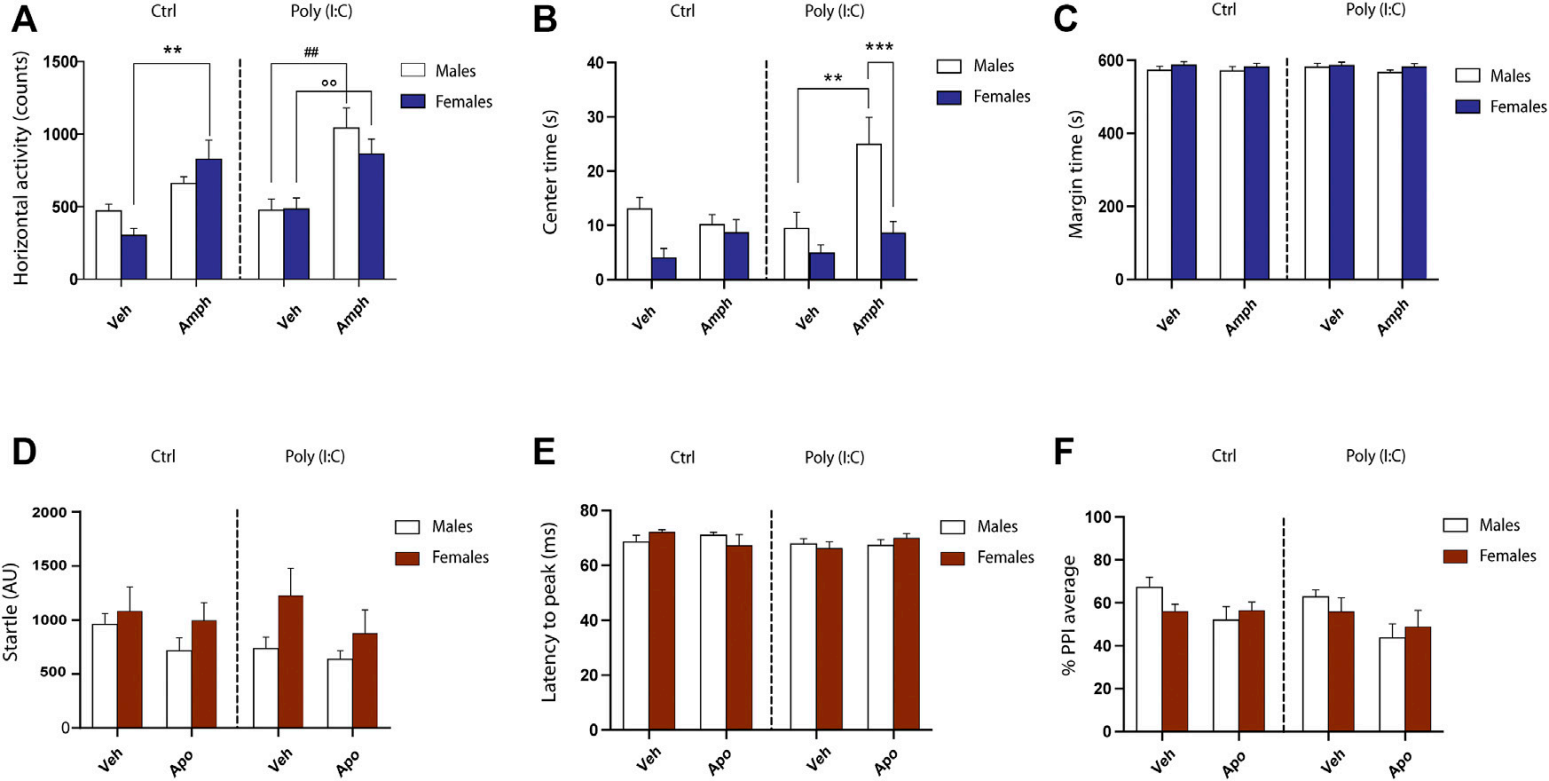
Behavioral phenotypes in F2 male and female poly (I:C) rats. **(A)** Counts of horizontal activity performed in the open field. **(B)** Time (seconds) spent in the center of the open field. **(C)** time spent in the periphery of the open field. Transgenerational effects of poly (I:C) on mean startle amplitude **(D)**, latency to peak **(E)** and prepulse inhibition (PPI) **(F)**. AU, arbitrary unit; ms, milliseconds. For further details, see the text. Data are expressed as means ± SEM. *p < .05 vs. rats treated with vehicle (Veh).

OF analyses revealed that poly (I:C) did not affect animals’ ethogram of male and female F2 progenies. As expected, at baseline the three-way ANOVA did not detect significant difference in motor activity as well as in the amounts of time spent in the central zone versus the outer zone of the field [motor activity, poly (I:C) X Sex interaction: F (1.51) = 0.42, *p* = .51, NS; Center time: F (1.51) = 1.74, *p* = .19, NS; Margin time: F (1.51) = 0.04, *p* = .84, NS] (Figure 3B,C). Conversely, the acute treatment with amphetamine elicited sex-dependent motor activation in F2 control and poly (I:C) offspring. Indeed, the three-way ANOVA analysis detected the interaction among sex X poly (I:C) X treatment [F (1.51) = 4.13 *p* < .04] (Figure 3A). Multiple comparisons indicated that, while the subthreshold dose of amphetamine-induced an increase in exploratory behavior in both F2- control and -poly (I:C) female rats, only male F2-poly (I:C) offspring exhibited a heightened motor activation in response to amphetamine administration (Figure 3A) [female-CTRL-SAL vs female-CTRL-AMPH, *p* < .01; female-poly (I:C)-SAL vs female-poly (I:C)-AMPH, *p* < .01; male-CTRL-SAL vs male-CTRL-AMPH, NS; male-poly (I:C)-SAL vs male-poly (I:C)-AMPH, *p* < .01]. In addition, albeit amphetamine exposure produced an increase in motor activity in both male and female F2- poly (I:C) progenies, its acute administration markedly changed the ethogram of F2- poly (I:C) male rats, as indexed by the higher amounts of time spent by the animals in the center of the arena (Figure 3B).

Consistent with F1 offspring (De Felice et al., 2019), male and female F2 progenies deriving from poly (I:C) offspring did not display any alterations in startle-related parameters, including mean startle amplitude [main effects of poly (I:C): F (1.52) = 0.35, *p* = .55, NS; interaction sex X poly (I:C): F (1.52) = 0.50, *p* = .48, NS] (Figure 3D), latency to peak [main effects of poly (I:C): F (1.52) = 1.34, *p* = .25, NS; interaction sex X poly (I:C): F (1.52) = 0.03, *p* = .86, NS] (Figure 3E), startle habituation [main effects of poly (I:C): F (1.52) = 0.01, *p* = .90, NS; interaction sex X Poly I:C: F (1.52) = 0.52, *p* = .47, NS] (data not shown). However, dissimilar to what observed by our group and others in F1 generation, poly I:C exposure failed to elicit significant deficits in PPI [main effects of poly (I:C): F (1.52) = 1.65, *p* = .20, NS], in both male and female F2 offspring [interaction sex X poly (I:C): F (1.52) = 0.09, *p* = .75, NS]. Finally, three-way ANOVA revealed that the acute treatment of a presynaptic low dose of apomorphine induced the disruption of PPI in F2 generation, independently from the variable poly (I:C) and/or sex (Figure 3F) [main effects of apomorphine: F (1.52) = 6.7 *p* < .05].

## 4 Discussion

Our study, the first to combine behavioral and electrophysiological characterization of the dopamine system in a maternal immune activation model across generations, reveals that poly (I:C) treatment in the F0 influences the dopamine system integrity not only in the F1 offspring (as previously demonstrated, De Felice et al., 2019; Luchicchi et al., 2016; Lecca et al., 2019) but also in the F2 progeny. In fact, male F2-poly (I:C) offspring showed 1) a marked decrease in the firing rate of spontaneously active dopamine cells in the VTA, 2) a heightened motor activation in response to a subthreshold dose of amphetamine, whereas, dissimilar to what observed by our group and others in the F1 generation, 3) failed to elicit significant deficits in PPI. It has been demonstrated that F1-poly (I:C) progeny showed marked alterations in key brain regions in the pathophysiology of schizophrenia, such as the ventral tegmental area (VTA), prefrontal cortex (PFC), nucleus accumbens (NAc), and hippocampus (Meyer et al., 2008b; Ito et al., 2010; Vuillermot et al., 2010). Moreover, maternal immune activation during pregnancy leads to abnormal neurodevelopmental trajectories interfering with postnatal brain maturation, brain anatomical changes, which might ultimately cause behavioral abnormalities in the offspring (Meyer et al., 2009; Piontkewitz et al., 2011; Gogos et al., 2020; Kreitz et al., 2020). In particular, an impaired dopamine system is considered a hallmark in schizophrenia (Abi-Dargham et al., 2000; Weinstein et al., 2017). Several studies found elevated dopamine levels in some brain regions, but lower or not changed levels were detected in other regions in MIA offspring (Winter et al., 2009; Abazyan et al., 2010; Kirsten et al., 2010; Giovanoli et al., 2013). Our group previously reported that males F1-MIA offspring but not females, exhibit behavioral deficits in sensorimotor gating, social interaction, and memory, as well as alterations in the mesocorticolimbic pathway (De Felice et al., 2019). Consistently, we also found that these outcomes are accompanied by a marked decrease of VTA dopamine neuron activity and a higher release of dopamine in the NAc (Luchicchi et al., 2016; De Felice et al., 2019; Lecca et al., 2019). A decreased frequency of discharge of spontaneously active dopamine neurons and a higher release of dopamine in the NAc, could be apparently discordant. However, previous studies demonstrated that firing frequency and release could not always be correlated, and a reduction in firing frequency might be adaptive to increased dopamine release (Floresco et al., 2003; Manta et al., 2013). Interestingly, in the present study, we confirmed a decreased discharge frequency in males but not females in the F2-poly (I:C) descendants, suggesting a transgenerational sex-dependent detrimental effect of MIA on the dopamine system. Next, we assessed the two major representative psychotic-like phenotypes, supported by an altered mesolimbic dopamine transmission, i.e., the potentiated sensitivity to amphetamine in the novel open field and deficits in PPI. Coherently, male F2-poly (I:C) offspring, but not female, exhibited a potentiated sensitivity to amphetamine mirrored by a heightened motor activation in response to amphetamine administration. This finding contrasts with previous studies that utilized the poly (I:C) in subsequent generations (Weber-Stadlbauer et al., 2021). In fact, Weber-Stadlbauer and colleagues found a blunted locomotor response to amphetamine in the F2. This discrepancy might be due to the profound differences in the neuropathological mechanisms between the two animal models (mice vs. rats). Moreover, administration of poly (I:C) (5 mg/kg vs. 4 mg/kg i. v.) to pregnant dams at a different time point (GD9 vs. GD 15) could influence the neurodevelopmental trajectories across generations. Another important difference is the amphetamine dose utilized: in this study, to unveil the heightened susceptibility of the mesolimbic dopamine system in the F2 generation, we chose to test a dose of amphetamine 10 times lower than those commonly reported in previous reports (Grilly and Loveland, 2001; Brudzynski et al., 2011; Higgins et al., 2020). Moreover, we and other groups showed that PPI alterations are not maintained across generations. In fact, in our previous study, the F1-poly (I:C) offspring displayed PPI dysfunctions (Luchicchi et al., 2016). Here, F2-poly (I:C) descendants did not show significant deficits in PPI. This finding is in accordance with previous transgenerational studies in the poly (I:C)-based MIA model (Bitanihirwe et al., 2010; Weber-Stadlbauer et al., 2017; Weber-Stadlbauer et al., 2021) that revealed how behavioral dysfunctions (e.g., PPI alterations, heightened motor activation in response to amphetamine) may differ across generations. The present data showing a reduction in firing rate of VTA dopamine neurons, potentiated sensitivity to amphetamine and lack of PPI dysfunctions corroborate previous findings on transgenerational detrimental effects of MIA on the dopamine system. Intriguingly, a previous study has suggested that in the MIA model male counterparts may also contribute to the aberrant offspring phenotype across generations (Weber-Stadlbauer et al., 2021). MIA elicits alterations in sperm DNA methylation, suggesting that this model is accompanied by epigenetic modifications in male germ cells. Thus, MIA may induce pathological traits across generations even in the absence of immune challenges (Weber-Stadlbauer et al., 2017; Berger et al., 2018; Weber-Stadlbauer et al., 2021). In addition, maternal care behavior could be important in the transgenerational effects of MIA. In fact, it has been previously demonstrated that maternal behavior elicited long-lasting inheritable effects on offspring brain development, function, and behavior (Ronovsky et al., 2017). Conversely, the lack of detectable alterations of maternal care behavior in other studies confirms how the interplay between maternal and paternal effects is complex (Weber-Stadlbauer et al., 2021). The use of poly (I:C) to trigger MIA leads to important advantages such as 1) the practical handling, avoiding the need to follow stringent biosafety precautions, 2) the time-limited immune response (24–48 h), that easily allows to study a precise time-point during pregnancy. However, with the current COVID-19 outbreak, it should be pointed out one of the major limitations of poly (I:C), as it does not reproduce the precise immunological scenario that occurs in the human environment following a viral infection. Viral infection models (e.g., influenza virus) would fit a higher level of construct and face validity for schizophrenia-related phenotype (Shi et al., 2003). On the other hand, it should be pointed out that the major impact of COVID-19 on the fetus during pregnancy might be related to the aberrant immune activation rather than the viral infection itself (De Biasi et al., 2021). Further limitations of our study are the use of a relatively small number of litters, which can lead to uncertain findings in multiparous species such as rats, single dose-testing in behavioral tests, the lack of the assessment of maternal care behavior as well as of paternal lineage on F2. Maternal care behavior could play a crucial role in the differences observed between F1 and F2 generation. We could hypothesize that F0 poly (I:C)-infected mothers display an “aberrant maternal care” (Ronovsky et al., 2017) towards their pups that could negatively impact the offspring behavior, which does not occur in F2 generation. With regard to VTA dopamine neuron activity, both F1 (De Felice et al., 2019; Lecca et al., 2019) and F2 generation display the same scenario, described by a reduction in firing rate. However, the lack of PPI deficits in the F2 generation, compared to the F1 generation, suggests that this phenotype across generations may need a second hit to unveil latent behavioral symptoms (e.g., challenge with apomorphine 0.125 mg/kg was too mild). Moreover, the PPI deficits discrepancy between F1 and F2 generation could be explained by the emergence of novel phenotypes in subsequent generation. Further studies are needed to assess if other behavioral domains are differently affected in the F2 generation, such as drug reward and/or decision making. Surprisingly, despite the primary involvement of the mesolimbic dopamine pathway in the MIA models, very few studies have addressed the impact of infections during pregnancy on addiction vulnerability in offspring.

Finally, as a potential explanation for the sex differences observed in our study, we cannot rule out the influence of sex steroids observed in F2 generation. Unfortunately, we neither assessed sex steroid hormones nor administered E2 or P4 antagonists to elucidate a potential protective effect of estradiol and progesterone in F2 females. To verify a potential role of female sex hormones, an interesting follow up study might be of investigating the electrophysiological and behavioral phenotypes in gonadectomized F2 female offspring exposed to Poly (I:C) infection during pregnancy.

In conclusion, this is the first study that highlights how MIA leads to sex-specific changes in mesolimbic dopaminergic activity and related behavioral readouts across multiple generations. Remarkably, the bias of dopaminergic transmission in male F2 progeny appears to selectively affect DA-dependent behavioral phenotypes, suggesting that the modifications of VTA dopamine neuron activity consequent of gestational infection may lead to transgenerationally pathological trajectories with relevance to discrete psychopathological traits later in life.

## Data Availability

The raw data supporting the conclusion of this article will be made available by the authors, without undue reservation.
