# Supplementary material for: Transgenerational Sex-dependent Disruption of Dopamine Function Induced by Maternal Immune Activation
Source: Front Pharmacol. 2022 Feb 8;13:821498. doi: 10.3389/fphar.2022.821498 (PMC8861303; doi:10.3389/fphar.2022.821498)
Supplement: Supplementary file 2 [file DataSheet1.DOCX]

Supplementary Material

**Supplementary Table 1. Number of animals and experimental design**

| Experiment | Figure | Number of dams | Number of litters | Number of animals |
| --- | --- | --- | --- | --- |
| Generation F1 | Fig.1 | See De Felice et al-. 2019 | See De Felice et al., 2019 | See De Felice et al., 2019; Lecca et al., 2019 |
| Generation F2 | Fig.1 | 3 ♀PIC from 3 different F1 litters were crossed with 3 ♂ Poly (I:C) from 3 different F1 litters. 3♀Veh from 3 different F1 litters.* F1 offspring allocated for breeding were littermates randomly selected following a recovery of at least 10 days after the completion of behavioral (PPI) testing. | Ctrl = 3 litters  Poly (I:C) = 3 litters | Ctrl n= 48 (26 ♂; 22 ♀)  Poly (I:C) n= 43 (22 ♂; 21 ♀)  Average litter size= 15,2  Total = 91 animals |
| Electrophysiology in vivo  F2 | Fig.2 |  |  | ♂ Ctrl = 7 (71 cells)  ♂ Poly (I:C) = 7 (72 cells)  ♀ Ctrl = 9 (72 cells)  ♀ Poly (I:C) = 8 (71 cells)  No more than 3 per litter  Total = 31 animals |
| Behavioral experiments F2  (Locomotor activity;  PPI)** | Fig.3 |  |  | ♂ Ctrl = 17 (no more than 3 per litter per each treatment)  ♂ Poly IC = 15 (no more than 3 per litter per each treatment)  ♀ Ctrl = 14 (no more than 3 per litter per each treatment)  ♀ Poly I:C = 14 (no more than 3 per litter per each treatment)  Total = 60 animals |

***** MIA males and females were mated together to produce an F2, combining maternal and paternal influences for the resulting F2.

****** The same animals used for both locomotor activity and PPI experiments.

**Supplementary Figure**

**Figure S1.**

Poly (I:C)-injected rat dams underwent a significant reduction in weight gain 24h after injection at GD15, when compared with vehicle injected dams. Student t-test, *** *P* < 0.0001,
